# Risk factor for elbow symptom manifestation in young baseball players with asymptomatic medial elbow abnormalities: a prospective cohort study

**DOI:** 10.1038/s41598-021-92570-9

**Published:** 2021-06-23

**Authors:** Hitoshi Shitara, Tsuyoshi Tajika, Takuro Kuboi, Tsuyoshi Ichinose, Tsuyoshi Sasaki, Noritaka Hamano, Fumitaka Endo, Masataka Kamiyama, Ryosuke Miyamoto, Kurumi Nakase, Atsushi Yamamoto, Tsutomu Kobayashi, Kenji Takagishi, Hirotaka Chikuda

**Affiliations:** Department of Orthopedic Surgery, Gunma University Graduate School of Medicine, 3-39-22, Showa, Maebashi, Gunma 371-8511 Japan

**Keywords:** Population screening, Paediatric research

## Abstract

Asymptomatic elbow abnormalities are relatively common in young baseball players, but the factors responsible are unclear. To prospectively identify risk factors related to symptom manifestation in asymptomatic elbow abnormalities, we recruited 573 baseball players (age: 7–14 years) at a pre-participation medical/physical examination in the preseason who were right-handed and had asymptomatic medial elbow abnormalities on ultrasound (US). Baseline preseason and postseason participant characteristics were assessed. A “symptomatic” elbow was defined as an elbow with medial elbow joint problems that prevented ball throwing for ≥ 8 days. After exclusions, 82 players were enrolled, of whom 22 (26.8%) developed a symptomatic elbow. In univariate analyses, the external and internal rotation strengths of the dominant shoulder were significantly greater in the symptomatic group than in the asymptomatic group (*P* = 0.021). Multivariate logistic regression analysis showed that the internal rotation strength of the dominant shoulder was a significant independent risk factor (odds ratio = 1.091, *P* = 0.027) for developing a symptomatic elbow. In young asymptomatic baseball players with abnormalities in the medial elbow region of the dominant arm on US, stronger preseason internal rotation strength of the dominant shoulder was a significant independent risk factor for the development of a “symptomatic” elbow.

## Introduction

Young baseball players are at high risk for elbow injuries^1–5^. It is believed that elbow injuries in high-school and college baseball players are caused by repeated microtrauma due to a high number of baseball throws during elementary or junior high school^5^. Repeated microtrauma and high-impact force to the medial elbow joint from throwing can cause medial epicondyle apophysitis, injury to the anterior bundle of the ulnar collateral ligament (UCL), or osteochondritis dissecans (OCD) of the humeral capitellum.

In baseball players, inability to throw the ball is mainly caused by elbow pain, discomfort, and/or instability. Prevention of elbow injuries comprises two phases; the goal of the first phase is to protect the elbow from anatomical failures, such as medial epicondyle apophysitis, UCL injury, and OCD of the humeral capitellum.

Recent studies have shown that elbow abnormalities seen on magnetic resonance imaging (MRI) were relatively common in 53.1% of young asymptomatic baseball players aged 9–13 years^6^, 65.0% of asymptomatic high school baseball players aged 15–19 years^7^, and 61.0% of asymptomatic professional baseball players^8^. These studies^6–8^ suggest that the detection of anatomical failures in the elbow based on symptoms alone is inaccurate because although a player may be asymptomatic, there could be underlying abnormalities. Thus, the second phase of prevention involves the coexistence of elbow joint anatomical failures and an asymptomatic condition that allows unhindered throwing. This concept seems impracticable because it is impossible to exclude baseball-related elbow abnormalities in competitive baseball players. Thus, a knowledge of the factors that lead to symptom presentation in asymptomatic players is important. However, prospective studies on baseball players with asymptomatic elbow abnormalities are limited.

In this study, we enrolled asymptomatic young baseball players to prospectively and comprehensively identify risk factors related to symptom manifestation in the dominant elbow. An asymptomatic state was defined as the absence of elbow pain at the time of preseason baseline assessment and the non-detection of elbow pain or medial elbow abnormalities on ultrasound (US).

## Results

### Participants

**Figure 1:**
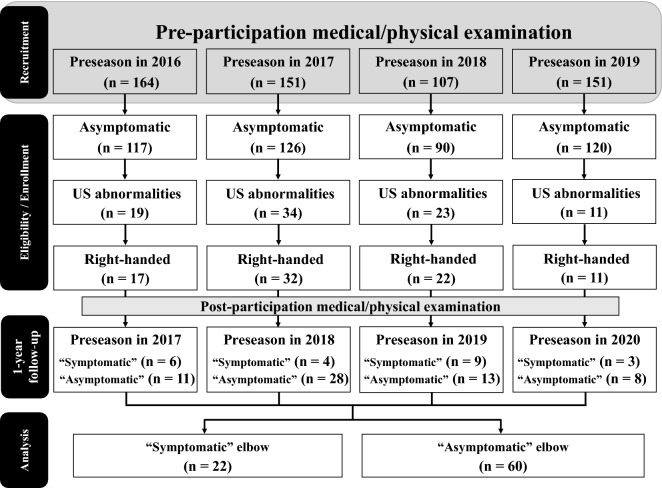
Flow chart of the players included in this study.

Of 573 players who participated in the annual pre-participation medical/physical examination in the preseason for 2 consecutive years, 453 players reported an asymptomatic dominant elbow joint. Medial elbow abnormalities were detected on US in 87 (19.2%) of the asymptomatic players. After excluding left-handed players (5 players), 82 were enrolled in this study, of whom 22 (26.8%) players had a throwing-related “symptomatic” elbow (Fig. 1).

### Baseline characteristics

The results of continuous and categorical data comparisons between the symptomatic (n = 22) and asymptomatic (n = 60) groups are shown in Tables 1 and 2, respectively.

**Table 1:** Demographic data (continuous data).

	Asymptomatic (N = 60)		Symptomatic (N = 22)		*P * value
	Mean	SEM	Mean	SEM	
Age (y)	10.6	0.2	10.8	0.3	0.684
Height (cm)	143.6	1.2	142.9	2.2	0.764
Weight (kg)	36.6	1.1	37.8	2.5	0.594
Practice days (days/week)	4.3	0.2	4.0	0.3	0.472
Duration of practice (hours/week)	21.6	0.6	19.6	1.5	0.157
Experience of catcher (year)	0.7	0.1	0.8	0.2	0.586
Experience of pitcher (year)	0.4	0.1	0.8	0.3	0.228
**ROM (degree)**					
**Elbow**					
Extension on the dominant side	5.6	0.7	5.3	1.0	0.809
Difference in extension	− 0.1	0.7	− 1.3	1.5	0.127
Flexion on the dominant side	139.4	0.4	138.0	0.7	0.338
Difference in flexion	− 2.4	0.6	− 3.3	0.9	0.423
**Shoulder**					
ABER on the dominant side	114.4	1.9	115.1	2.1	0.845
Difference in ABER	5.9	1.5	8.8	2.7	0.322
ABIR on the dominant side	44.1	2.2	42.5	2.3	0.690
Difference in ABIR	− 7.6	1.9	− 9.0	2.5	0.695
Total arc on the dominant side	158.5	2.9	157.6	2.8	0.863
Difference in total arc	− 1.7	2.3	− 0.2	2.5	0.711
HA	14.0	1.5	19.5	2.4	0.057
Difference in HA	− 7.9	2.0	− 6.0	2.4	0.605
**Hip**					
SLR on the back leg	69.2	1.0	66.9	4.0	0.588
SLR on the front leg	68.0	1.2	71.3	1.9	0.158
Flexion on the back leg	124.0	1.3	121.4	3.8	0.428
Flexion on the front leg	124.6	1.3	124.2	2.1	0.854
Supine IR on the back leg	40.9	2.2	43.6	5.8	0.595
Supine IR on the front leg	41.2	1.7	40.8	4.9	0.912
Supine ER on the back leg	49.9	1.6	49.4	4.4	0.883
Supine ER on the front leg	52.1	1.5	51.6	2.5	0.887
PIR on the back leg	51.3	1.3	51.2	2.2	0.983
PIR on the front leg	52.2	1.5	51.4	1.8	0.766
PER on the back leg	55.6	1.4	56.6	2.1	0.699
PER on the front leg	53.0	1.4	53.0	2.0	0.998
**Knee**					
Extension on the back leg	− 0.1	0.2	0.0	0.0	0.839
Extension on the front leg	− 0.1	0.2	0.0	0.0	0.817
Flexion on the back leg	150.2	1.0	148.8	0.9	0.597
Flexion on the front leg	148.4	1.0	148.5	1.9	0.970
**Ankle**					
Dorsiflexion on the back leg	21.3	1.1	19.1	1.6	0.305
Dorsiflexion on the front leg	20.0	1.1	18.4	1.4	0.412
Plantarflexion on the back leg	52.9	1.0	51.1	1.6	0.361
Plantarflexion on the front leg	53.4	1.1	50.9	1.6	0.222
**Strength**					
**Hand**					
Grip on the dominant side (kgw)	17.0	0.8	16.8	1.9	0.927
Grip ratio	1.2	0.1	1.0	0.0	0.317
**Shoulder**					
PER on the dominant side (kgw)	11.8	0.7	14.7	1.3	0.039
PER ratio	1.1	0.0	1.0	0.0	0.621
PIR on the dominant side (kgw)	12.6	0.7	16.3	1.6	0.021
PIR ratio	1.0	0.0	1.0	0.0	0.381
PER/PIR ratio on the dominant side	1.0	0.0	0.9	0.0	0.679

* *P* < 0.05. SEM: standard error of the mean.

ABER, 90° abduction and external rotation; ABIR, 90° abduction and internal rotation; Total arc, ABER + ABIR; HA, horizontal adduction; ROM, range of motion; ROM difference, ROM of the dominant elbow minus ROM of the non-dominant elbow; SLR, straight-leg raising angle; ER, external rotation; IR, internal rotation; PER, prone external rotation; PIR, prone internal rotation; Strength ratio, dominant/non-dominant.

**Table 2:** Demographic data (categorical data).

	Asymptomatic (N = 60)		Symptomatic (N = 22)		*P *value
	n	(%)	n	(%)	
**Baseline characteristics**					
**Sex**					0.796
Boys	58	(96.7)	21	(95.4)	
Girls	2	(3.3)	1	(4.5)	
**Position**					0.297
Pitcher	4	(6.7)	4	(18.2)	
Catcher	6	(10.0)	2	(9.1)	
Fielder	50	(83.3)	16	(72.7)	
**Pitching experience**					0.657
Yes	24	(40.0)	10	(45.5)	
No	36	(60.0)	12	(54.5)	
**Catching experience**					0.956
Yes	16	(26.7)	6	(27.3)	
No	44	(73.3)	16	(72.7)	
**Numbers of full-power throws /week**					0.144
None	15	(25.0)	1	(4.5)	
≤ 50	20	(33.3)	8	(36.4)	
51–100	18	(30.0)	11	(50.0)	
> 100	7	(11.7)	2	(9.1)	
**Presence of pain**					
**Shoulder on the dominant side**					0.386
Yes	2	(3.3)	0	(0.0)	
No	58	(96.7)	22	(100.0)	
**Back**					-
Yes	0	(0.0)	0	(0.0)	
No	60	(100.0)	22	(100.0)	
**Lower back**					0.113
Yes	1	(1.7)	2	(9.1)	
No	59	(98.3)	20	(90.9)	
**Hip on each side**					-
Yes	0	(0.0)	0	(0.0)	
No	60	(100.0)	22	(100.0)	
**Knee on each side**					0.722
Yes	4	(6.7)	1	(4.8)	
No	56	(93.3)	21	(95.2)	
**Ankle on each side**					-
Yes	0	(0.0)	0	(0.0)	
No	60	(100.0)	22	(100.0)	
**Elbow on the dominant side**					
**Elbow valgus stress test**					0.454
Yes	1	(1.7)	1	(4.5)	
No	59	(98.3)	21	(95.5)	
**Elbow US findings on the dominant side**					
**Capitellum**					0.493
Normal	57	(95.0)	20	(90.9)	
Abnormal	3	(5.0)	2	(9.1)	
**Medial epicondyle**					0.081
Type 1	0	(0.0)	0	(0.0)	
Type 2	26	(43.3)	7	(31.8)	
Type 3	29	(48.3)	9	(40.9)	
Type 4	5	(8.3)	6	(27.3)	

US, ultrasound.

Shoulder prone external rotation (PER) and prone internal rotation (PIR) strengths on the dominant side were significantly greater in the symptomatic group than in the asymptomatic group. No significant differences in age; height; weight; practice days; practice duration; catching and pitching experience; sex; current field position; number of full-power throws per week; presence of shoulder, back, lower back, hip, knee, or ankle pain; elbow valgus stress test findings; US findings in the dominant elbow; range of motion (ROM) of the elbow, shoulder, hip, knee and ankle; dominant side grip strength; strength ratios of grip and shoulder PER and PIR; and the PER/PIR ratio was observed between the groups.

### Logistic regression analysis

Based on the results of univariate analyses (*P* < 0.05), we selected the PER and PIR strength of the dominant shoulder for logistic regression analysis.

Logistic regression analysis showed that the PIR strength of the dominant shoulder was a significant independent risk factor (odds ratio [OR] = 1.091, 95% confidence interval [CI] = 1.010 − 1.179, *P* = 0.027; Table 3) for dominant elbow symptom manifestation. Assuming that the mean PIR strength of the dominant shoulder in the symptomatic group was 3.7 kgw greater than that of the dominant shoulder in the asymptomatic group, the adjusted OR was calculated to be 0.726. This OR indicates that players in the symptomatic group may experience a 27.4% reduction in the risk of a symptomatic elbow if they decrease the PIR strength of the dominant shoulder by 3.7 kgw.

**Table 3:** Results of logistic regression analysis.

Explanatory variable	Odds ratio	95% CI	*P* value
Shoulder PIR strength on the dominant side	1.091	1.010–1.179	0.027*

CI, confidence interval; PIR, prone internal rotation.

* *P* < 0.05.

### Correlation between shoulder PIR strength and physical examination findings of the lower limbs

Because shoulder PIR strength was considered a risk factor, we performed a correlation analysis between shoulder PIR strength and physical examination findings of the hip, knee, and ankle, as the upper flow of the systemic kinetic chain of the shoulder. There was a significant negative correlation between shoulder PIR strength on the dominant side and hip ROM of PIR on the back leg (r = − 0.319, *P* = 0.025). No further correlations were found between shoulder PIR strength on the dominant side and any other physical examination finding of the hip, knee, or ankle.

### Progression of medial elbow abnormalities

There was no significant relationship between the progression of medial elbow abnormalities seen on US and elbow symptom manifestation (*P* = 0.283; Table 4).

**Table 4:** Relationship between the progression of medial elbow abnormalities on ultrasound and elbow symptom manifestation.

	Asymptomatic (N = 60)		Symptomatic (N = 22)		*P *value
	n	(%)	n	(%)	
**Progression in medial elbow findings on ultrasound**					0.283
Yes	10	(16.7)	6	(27.3)	
No	50	(83.3)	16	(72.7)	

### Post-hoc power analysis

Post-hoc power analysis for the univariate analysis revealed a power of 1.0 for each significantly different factor.

## Discussion

The most important finding of this study is that greater preseason PIR strength of the dominant shoulder was a significant independent risk factor for symptom manifestation. Players in the symptomatic group may achieve a 27% risk reduction in the manifestation of symptoms if they decrease the PIR strength of their dominant shoulder such that it becomes equal to that of players in the asymptomatic group. There was a significant negative correlation between shoulder PIR strength and hip ROM of internal rotation. Furthermore, there was no significant relationship between the progression of medial elbow abnormalities seen on US and symptomatic elbow manifestation. To our knowledge, this prospective study is the first to establish that greater preseason PIR strength of the dominant shoulder is a significant independent risk factor for symptom development in young asymptomatic baseball players with US-detected medial elbow abnormalities. Furthermore, this study demonstrates that the progression of medial elbow abnormalities on US may not manifest as a symptomatic elbow.

Pennock et al.^9^ prospectively investigated 26 asymptomatic Little League players, aged 10 to 13 years, and demonstrated that abnormal MRI-detected elbow findings were significantly associated with year-round play (playing ≥ 8 months a year) and private coaching. Furthermore, the authors reported a significant association between a history of pain and year-round play, but no significant correlation was noted between abnormal MRI findings and playing position, baseball experience, history of elbow pain, or compliance with throwing guidelines. The authors additionally investigated the same participants in the next season and reported that 48% of players showed MRI-detected abnormalities on the dominant elbow and 28% of these players experienced arm pain during the season^10^. In that study, year-round play was a significant risk factor for postseason MRI-detected elbow abnormalities, and no significant association was observed between postseason elbow abnormalities on MRI and number of games, position, pitch counts, pitch innings, pitch types, private coaching, or any physical examination findings, including the ROMs of the shoulder and elbow.

Garcia et al.^8^ retrospectively investigated 41 Major League Baseball pitchers who had no prior injured list placement; 39% of these patients had normal findings on MRI of the elbow and 61% had abnormal findings, at a baseline assessment. The authors defined “injured” as being placed on the injured list, while the reverse case was defined as “healthy.” The authors demonstrated that MRI findings of posteromedial impingement, UCL heterogeneity, and humeral-side partial UCL tears were significantly correlated with future placement in the injured list for elbow abnormalities. Compared to previous study findings, the current findings seem more reliable because of the larger sample size and homogeneous population in the present study^8,9^.

Although different risk factors are more likely to trigger symptoms in patients with newly developed elbow abnormalities than in those with existing elbow abnormalities, previous studies^8,9^ enrolled baseball players with and without MRI-detected elbow abnormalities in the baseline assessment. We believe that for a study to detect risk factors for a “symptomatic” elbow in asymptomatic baseball players with elbow abnormalities, it should include only players with US-detected elbow abnormalities and no elbow pain (i.e. no history of elbow pain and no current elbow pain) at the baseline assessment. Because participants with a history of elbow pain were not excluded in the previous study^9^, it is difficult to ascertain the risk factors for symptomatic elbow abnormalities based on the study findings. Moreover, the participants in that study^9^ may have only been temporarily asymptomatic at the time of baseline assessment. To avoid this bias, we included only asymptomatic players with no history of elbow pain. To our knowledge, this is the first prospective study to include only asymptomatic (i.e., asymptomatic at the time of enrollment, with no history of elbow pain) baseball players with medial elbow abnormalities.

We demonstrated that higher PER and PIR strengths of the dominant shoulder were significantly associated with symptom manifestation in dominant elbows with abnormal findings on US. We did not ask the participants whether they were receiving private coaching because hiring a private coach was not a common practice. Although the baseball federation in the prefecture where the present study was performed recommends off-season play, most young baseball players in our country play year-round. All participants in this study played ≥ 9 months a year, which exceeds the 8 months a year duration in the definition of “year-round play” in previous studies^9,10^.

Holt et al.^11^ prospectively evaluated the progression of MRI-detected elbow abnormalities in 26 Little League players (aged 12 to 15 years) to determine whether pitchers, catchers, or year-round players (defined as ≥ 8 months per year) with continued play would have more severe and progressive elbow abnormalities on MRI at the 3-year follow-up. The authors found that 57.7% of players displayed dominant elbow MRI pathology at the 3-year follow-up, with 80.0% of these patients developing new or progressive abnormal findings, compared to the findings of prior MRI studies. However, the authors did not find significant correlations between progressive abnormal MRI findings and throwing history (including years of play, primary position of play, months of play per year, and physical examination findings, such as shoulder ROM, shoulder strength, and shoulder and elbow instability). Holt et al.^11^ also reported that year-round play was a significant predictor of positive MRI findings at 3 years; however, the MRI findings included both new lesions and pre-existing lesions, which do not reflect the progression of abnormal MRI findings. Thus, it remains unclear which factors influence the progression of elbow abnormalities on US or MRI. However, for baseball players, determining the risk factors for symptom manifestation in the dominant elbow seems more important than determining the factors that are related to the progression of elbow abnormalities. In the present study, we prospectively demonstrated the lack of a significant association between symptom manifestation in the dominant elbow and the progression of elbow abnormalities on US.

Harada et al.^4^ prospectively investigated baseball players, aged 9–12 years, and demonstrated that greater shoulder muscle strength, evaluated with the participant in a sitting position (external rotation > 8.2 kg; internal rotation > 10.2 kg), was a risk factor for elbow injury. Shitara et al.^12^ reported that the ratio of dominant-side to non-dominant-side PER was an independent risk factor for shoulder and elbow injury in high school baseball pitchers. Byram et al.^13^ reported that in professional baseball pitchers, PER strength was significantly associated with throwing-related shoulder and elbow injuries that required surgical intervention. Consistent with the findings of a similar previous study^4^, our results showed that greater PER and PIR strengths on the dominant side were significantly associated with symptom manifestation in dominant elbows with abnormal findings on US. Aguinaldo and Chambers ^14^ demonstrated that elbow valgus torque in pitchers who rotated their upper trunk before front foot contact was significantly greater than that in pitchers who rotated their upper trunk after front foot contact. This suggests that players tend to generate more internal rotation torque in the shoulder of the throwing arm to compensate for rotational energy loss from a poor kinetic chain^14,15^.

Hamano et al. demonstrated that preseason limited ROM of the hip with 90° flexed external rotation in the front leg was a risk factor for shoulder/elbow pain in the playing season^16^. In a cross-sectional study, these authors additionally reported that hip external rotation ROM on the back leg was significantly lower in injured high school baseball pitchers than in non-injured pitchers^17^. A previous prospective study showed that preseason decreases ROM in flexion bilaterally and internal rotation on the back leg were risk factors for shoulder and elbow injuries in elementary and junior high school baseball players^18^. Hip ROM has not been established as a risk factor for baseball-related injury. In this study, although hip ROM limitation was not a risk factor, there was a significant negative correlation between shoulder PIR strength on the dominant side and hip PIR of the back leg.

Based on the findings of biomechanical studies^14,15^ and our study results, which indicate that greater shoulder PIR strength was significantly correlated with limited hip ROM of PIR on the back leg, the greater shoulder strength in the symptomatic group may be a compensation for energy transmission loss due to poor sequential body motion.

### Limitations

This study had several limitations. First, we included players who participated in the annual pre-participation medical/physical examination in the preseason for 2 consecutive years. This participant selection may have been biased, and this may have affected the results. Second, all participants were year-round players because most young competitive baseball players in our country play baseball year-round. Therefore, our results may not be generalizable to players in other countries. Third, the OR of shoulder internal rotation strength was relatively low (1.091). Thus, it might be a weak risk factor. However, we believe the finding about a 27.4% risk reduction in symptomatic players is meaningful. Fourth, we did not evaluate the maximum ball speed and the increasing rate of height and body weight during the season, as these data could not be acquired from the preseason medical/physical examination. Those factors might be related to shoulder PIR strength. However, we believe that the aforementioned increasing rate of height and body weight do not greatly impact our results because there were no differences in height and weight at baseline between the groups. Finally, there may have been some recall bias because the participants’ history, including pain and player position, were obtained retrospectively.

### Conclusion

In young asymptomatic baseball players with US-detected elbow abnormalities on the dominant side, greater preseason PIR strength of the dominant shoulder was a significant independent risk factor for a symptomatic dominant elbow. Clinicians should carefully assess players with greater shoulder strength to determine the cause of energy transmission loss, such as limitation in hip ROM of internal rotation on the back leg, rather than suggest a reduction in shoulder strength. Furthermore, our study findings demonstrate that the progression of elbow abnormalities on US may not be associated with the development of subjective symptoms. Thus, clinicians should note the gradual progression of elbow abnormalities through regular examination, such as annual medical/physical examination.

## Methods

### Participants

From 2016 to 2019, we recruited competitive league baseball players aged 7–14 years, who played for 9 months a year (March−November), at annual medical/physical examinations. Based on the inclusion criteria in previous studies^12,19^, we included players who (1) participated in annual pre-participation medical/physical examination in the preseason for 2 consecutive years (i.e. either 2016 and 2017, 2017 and 2018, 2018 and 2019, or 2019 and 2020), (2) had participated in preseason practice as an active player at the first pre-participation medical/physical examination; (3) had no restrictions in baseball activities, such as throwing, running, and batting, at the first pre-participation medical/physical examination; (4) showed medial elbow abnormalities in the dominant side on US, and (5) were right-handed, because side-to-side differences in glenohumeral external rotation angle and humeral torsion angle were significantly different between right-handed and left-handed pitchers among young baseball players^20^. The exclusion criteria^12,19^ were (1) past or current history of elbow pain on the dominant side at the first pre-participation medical/physical examination, (2) prior injuries (e.g., fracture) of the throwing arm, and (3) inability to play baseball because of foot, ankle, knee, hip, spine, shoulder, or elbow problems at the first pre-participation medical/physical examination. Prior to enrollment, we obtained informed consent from the participants’ parents. All procedures were conducted in compliance with relevant regulations and guidelines. The institutional review board of Gunma University Hospital (identification number, 1003) approved this study.

### Medical/physical examination

As in previous reports^12,19^, in the current study, pre-participation medical/physical examination in the preseason was performed as baseline medical examinations to evaluate the preseason condition of the participants’ shoulders and elbows. To avoid confirmation bias, the examiners were unaware of the participants’ hand dominance. We evaluated (1) age; (2) height; (3) weight; (4) sex; (5) current position; (6) pitching and catching experience; (7) number of full-power throws per week during the season, (8) presence of shoulder, back, lower back, hip, knee, and ankle pain; (9) ROMs of the bilateral elbow, shoulder, hip, knee, and ankle; (10) grip and shoulder strength; (11) elbow valgus stress test; and (12) US evaluation of the elbow. Participants, coaches, and parents were asked to fill out a questionnaire regarding the players’ age, sex, current position, pitching and catching experience, number of full-power throws per week during the prior season, and the presence of pain in the shoulder, back, lower back, hip, knee, and ankle.

### ROM measurement

The intra-rater validity and reliability of ROM measurements using a digital protractor have been established^19^. All passive ROM measurements were conducted by certified orthopedic surgeons using a digital protractor. During the measurements, participants were instructed to lie supine or prone on an examination bed and to relax.

#### Elbow and shoulder ROM

Based on procedures used in previous studies^12,19^, passive elbow ROM in flexion and extension, passive shoulder ROM in horizontal adduction (HA), and 90° abduction and external rotation (ABER) and abduction and internal rotation (ABIR) were measured bilaterally with the participant in a supine position.

#### Hip ROM

Based on previously reported procedures^21^, we measured hip ROM in flexion and internal/external rotation with the hip flexed 90° and the participant in a supine position, and hip ROM in internal/external rotations of the extended hip with the participant in a prone position. The straight-leg raising angle was measured using a standard technique^22^, with the participant in a supine position.

#### Knee

Based on standard procedures, passive bilateral knee ROM in flexion and extension was measured, with the participant in a supine position.

#### Ankle

As described previously^23^, participants were asked to lie in a supine position with the hip, knee, and ankle joints in a neutral position of flexion/extension and inversion/eversion. Subsequently, the dorsiflexed and plantarflexed ankle and foot joints were measured.

### Shoulder strength measurement

The intra-rater validity and reliability of shoulder strength measurements obtained using hand-held dynamometers have been established^19^. As in the literature^12,19^, participants were asked to lie in a prone position with the humerus abducted 90° and the elbow flexed 90°. Next, the PER and PIR strengths of the prone shoulder were measured bilaterally by certified orthopedic surgeons, using the PowerTrack II Commander hand-held dynamometer.

Each measurement was repeated three times and recorded. The median value of the data was analyzed. The dominant to non-dominant ratios of PER and PIR strengths and the PER/PIR ratio on the dominant side were calculated for each participant.

### Grip strength

The bilateral grip strength was measured using a digital dynamometer via a standardized position recommended by the American Society of Hand Therapists^24^.

### Elbow valgus stress test

The intra-rater validity and reliability of the elbow valgus stress test have been established^12^. Briefly, participants were instructed to lie in a supine position on an examination bed, with the shoulder abducted 90° and slightly extended horizontally, the forearm supinated, and the elbow flexed 90°. A certified orthopedic surgeon evaluated medial elbow laxity using the milking maneuver^25^, which involves generating a valgus force by pulling the participant’s thumb. We defined a positive outcome as medial joint pain, laxity, no firm end point, or a participant complaint of apprehension.

### US evaluation of the elbow joint on the dominant side

The intra-rater validity and reliability of US evaluation of the elbow joint have been established^26,27^. A multifrequency 13-MHz linear array transducer was used by certified orthopedic surgeons with > 10 years of experience in musculoskeletal US to determine elbow abnormalities^4,28,29^.

**Figure 2:**
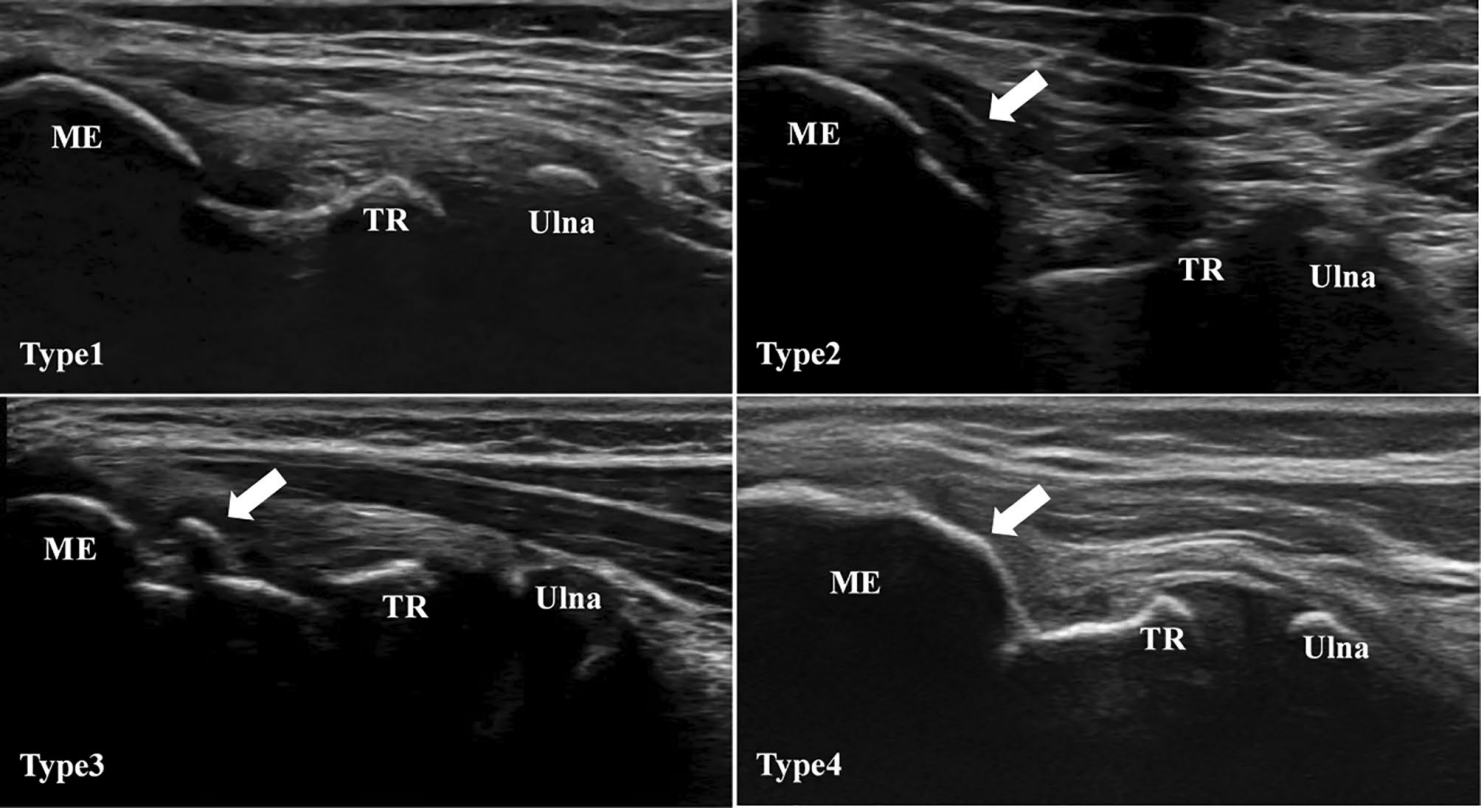
Longitudinal ultrasonography images of the medial elbow joint. Type 1: normal (upper left); Type 2: arrow indicates a blurred image of the MCL at the attachment to the medial epicondyle (upper right); Type 3: arrow indicates segmentation of the medial epicondyle at the attachment of the MCL (lower left); Type 4: arrow indicates protrusion of the medial epicondyle at the attachment of the MCL (lower right). MCL, medial collateral ligament; ME, medial epicondyle; TR, trochlea U; ultrasound.

According to a previous report^30^, the assessment of the medial elbow joint was defined as follows: (Type 1)—normal; (Type 2)—blurred image of the medial collateral ligament (MCL) at the attachment to the medial epicondyle; (Type 3)—medial epicondyle separation or segmentation at the attachment of the MCL; and (Type 4)—medial epicondyle protrusion at the attachment of the MCL (Fig. 2**)** ^29^. The presence of medial elbow abnormalities, defined as Types 2–4, was an inclusion criteria in this study, as previously described. We also defined OCD of the capitellum as an irregularity of the subchondral bone of the capitellum^31^. Decisions during medical examinations, including decisions regarding the progression of elbow abnormalities, were based on consensus among the three certified orthopedic surgeons. Disparate opinions between the surgeons were resolved by further discussions.

### Symptom tracking

Although it is important to evaluate the pathophysiology of elbow injury to determine the appropriate treatment, the ability to throw a baseball without discomfort is a more important need for players. Thus, we defined elbows as “symptomatic” when medial elbow abnormalities, such as discomfort, result in the inability to throw a baseball for ≥ 8 days^12,19,32^. We excluded injuries that were sustained when the player was hit by a ball, resulting in collision with another player, or injuries that resulted from a fall.

### Progression of medial elbow abnormalities

We evaluated the medial elbow joint on the dominant side using the same US machine pre- and postseason. We defined “progression” as changes in US findings between pre- and postseason assessments, from Type 2 to Type 3 (newly developed segmentation of the medial epicondyle at the attachment of the MCL) or from Type 4 to Types 2 or 3 (newly developed MCL injury at the attachment to the medial epicondyle or segmentation of the medial epicondyle at the attachment of the MCL). As described above, the decision was based on the consensual analysis of the orthopedic surgeons.

### Statistical analyses

To determine the sample size for the logistic regression analysis in this study, a prior statistical power analysis was performed using G*Power 3.1.9.4 (Heinrich Heine University, Dusseldorf, Germany)^33^, which indicated that 70 participants were required to detect statistical significance, based on a statistical power of 80% at an alpha level of 0.05 (i.e., assumptive incidence rate = 20%; OR = 2.5)^34^.

Regarding the results of the univariate analyses of the baseline characteristics, continuous variables are presented as mean ± standard error of the mean, and categorical variables are presented as number (n) and percentage (%). Group differences in baseline characteristics between the symptomatic and asymptomatic groups were evaluated using the Mann–Whitney *U* test for continuous data and the chi-square test for categorical data. After adjusting for significant variables determined from the univariate analyses, a logistic regression analysis was performed to detect risk factors for symptom manifestation in players with asymptomatic US-detected medial elbow abnormalities. We selected explanatory variables for the model based on the results of univariate analyses (*P* < 0.05). To confirm whether there was energy transmission loss due to a poor systemic kinetic chain during the pitching motion, a correlation analysis of the identified independent risk factor and physical examination findings in the upper flow of the systemic kinetic chain (e.g., risk factor = shoulder function; upper flow of shoulder = trunk, hip, knee, and ankle) was performed. To test the relationship between symptom development and the progression of medial elbow abnormalities, a chi-square test was performed. Finally, we performed a post-hoc power analysis to verify the statistical power of this study using G*Power 3.1.9.4^33^.

All tests were two-sided (*P* = 0.05). We used the Statistical Package for the Social Sciences version 25 (IBM Japan, Ltd., Tokyo, Japan) for all statistical analyses, without sample size calculation and post-hoc power analysis.

### Consent to participate

Informed consent was obtained from the participants’ parents.

### Consent to publish

Consent for publication was obtained from the participants’ parents.

## Data Availability

Data supporting the findings of this study are available on request from the corresponding author. Data are not publicly available because they contain information that could compromise the privacy of the research participants.
